# Does screening for vaginal infection have an impact on pregnancy rates in intracytoplasmic sperm injection cycles?

**DOI:** 10.4274/tjod.56563

**Published:** 2016-03-10

**Authors:** Özlem Eldivan, Özlem Evliyaoğlu, Ebru Ersoy, Gönül Aksu, Serdar Dilbaz, Ümit Göktolga

**Affiliations:** 1 Etlik Zübeyde Hanım Women’s Healthcare Education and Research Hospital, Clinic of Obstetrics and Gynecology, Ankara, Turkey; 2 Etlik Zübeyde Hanım Women’s Healthcare Education and Research Hospital, Clinic of Microbiology, Ankara, Turkey

**Keywords:** Infection, screening, culture, assisted reproduction, pregnancy rates

## Abstract

**Objective:**

Assisted reproduction techniques have become widespread worldwide. Considering their costs, physicians endeavor to improve pregnancy rates. Infections are one of the disrupting problems in this arena. We aimed to investigate the effects of screening for vaginal infection on pregnancy rates in intracytoplasmic sperm injection cycles.

**Materials and Methods:**

One hundred twenty patients randomized into two groups for this study. Patients were screened for vaginal infections in group 1, and no screening was performed in group 2. The assisted reproduction outcomes were investigated and compared between the two groups.

**Results:**

There was no significant difference between ages, or durations and causes of infertility of patients who conceived and of those who did conceive. Forty-five patients in group 1, and 40 patients in group 2 reached the embryo transfer stage. The rates of conception were 23.5% (n=4) in culture-positive patients (n=17), and 42.9% (n=12) in culture-negative patients (n=28) in group 1. There was no significant difference among patients who were not screened, screen-positive, and screen-negative, in terms of pregnancy rates. None of the patients had Neisseria gonorrhoeae or Trichomonas vaginalis. Bacterial vaginosis was detected in 13 patients, and both bacterial vaginosis and *Chlamydia trachomatis* were detected in 4 patients. Three of 4 patients who conceived screen-positive and 8 of 12 patients who conceived screen-negative delivered healthily at term.

**Conclusion:**

No significant difference was found between patients who were sampled for culture and patients who were not sampled in terms of pregnancy rates. Also, no difference was found between the patients who were culture-negative and patients who were treated with antimicrobials after a culture positive result. Further larger studies are warranted to clarify this issue.

## PRECIS:

No significant difference was found between patients who were sampled for culture and those who were not sampled in terms of pregnancy rates in intracytoplasmic sperm injection cycles.

## INTRODUCTION

Significant increase in the number of couples seeking treatment for infertility. Their increased use has been associated with significant economic costs because they are expensive procedures. As stated in a study in the Netherlands, costs per cycle started at €2381 and €2578 for in vitro fertilization (IVF) and Intracytoplasmic sperm injection (ICSI), respectively^(1)^. The pregnancy rate in IVF programmes remains about 20-50% inspite of the high rate of successful fertilization. This has led to the proposition that additional factors critical for the implantation process must be limiting^(2)^. There is growing evidence that cervical-vaginal flora may strongly influence pregnancy rates. A few studies showed that the presence of microbial flora of the cervix on embryo transfer (ET) catheter was associated with poor IVF-ET outcomes^(3,4,5)^. *Chlamydia trachomatis (C. trachomatis)* and *Neisseria gonorrhoeae (N. gonorrheae)* are the most prevalent sexually-transmitted bacterial infections worldwide. Although most genitourinary tract infections of *C. trachomatis* in women are asymptomatic, it is a major public health problem because of the recent rises in the reported number of cases and the severe reproductive morbidity that results from untreated infections and associated costs to the health services^(6)^. However, bacterial vaginosis (BV) is being increasingly implicated in upper genital tract infections in women^(7)^. *Trichomonas vaginalis* is a far more prevalent sexually-transmitted infection than either *Chlamydia trachomatis* or *Neisseria gonorrhoeae*, yet in stark contrast, little attention is paid to trichomoniasis. It is suggested that it can reduce the chances of conception from both female and male factors and should be considered in the diagnostic tests of infertile couples^(8)^.

The primary objective of our study was to investigate the effect of screening for vaginal infections on pregnancy success in patients undergoing ICSI in our clinics.

## MATERIALS AND METHODS

Women undergoing IVF at the Assisted Conception Unit of Etlik Zubeyde Hanim Women’s Health Education and Research Hospital between April 2009 and June 2009 were recruited for this case-control study. Ethical approval for the study was obtained from the local ethics committee (approval date/number: 13.03.2009/7) and all participants gave their written consent to participate. The research was completed in accordance with the Declaration of Helsinki^(9)^.

Women who had basal serum levels of follicle-stimulating hormone <10 mIU/L, prolactin, free triiodothyronine (fT_3_), free thyroxine (fT_4_), and thyroid-stimulating hormone levels within the normal range, aged <38 years, and asymptomatic for leukorrhea underwent a long luteal GnRH-analog protocol. Patients who had received antibiotic treatment during the previous three months were excluded from the study. All the enrolled women were informed that they had been investigated for sexually-transmitted microorganisms and had been thoroughly tested in accordance with our clinic protocol, which included a baseline early follicular phase endocrine profile, and baseline transvaginal ultrasonography including antral follicle count. Age, cause, and duration of infertility was recorded. On completion of the investigations, 60 patients were randomly assigned to the study group (group 1) and 60 patients to the control group (group 2). Randomization was based on computer-generated codes. In group 1, patients were screened for vaginal infections and no screening was performed in group 2. The women entered the study on the day of gonadotropin initiation. The women were treated with a conventional superovulation regimen of pituitary down-regulation followed by stimulation with gonadotropins.

Specimens were collected immediately after menses. Cultures for *N. gonorrhea* and *C. trachomatis*vaginosis and Trichomonas were collected separately from the endocervix and vaginal cultures were taken to investigate the presence of bacterial vaginosis and *Trichomonas vaginalis.* A non-lubricated bivalve speculum was inserted and the vaginal walls and posterior fornix were sampled using two sterile cotton swabs. One swab was rolled on a glass slide for Gram staining, and the flora were investigated for BV using Nugent’s criteria. One swab was placed in aerobic transport media. A second droplet of discharge was mixed with saline on another slide for the wet mount. The wet mount was immediately examined to detect motile Trichomonas trophozoites. *C. trachomatis* was investigated via QuickVue test using as enzyme immunoassay (EIA). QuickVue is a rapid chlamydial antigen capture assay based on genus-specific murine monoclonal antibody. Specimens for N. gonorrhoeae were collected from the endocervix as described above. The specimens were immediately plated directly onto modified Thayer-Martin medium.

The time between obtaining the culture result and the day for oocyte pick up in each patient was at least 5 days because the cultures and gram stain were collected at the beginning of the IVF cycle after menses.

Patients were treated with proper antimicrobials according to the recommendations of the Center for Disease Control and Prevention (CDC) when organisms are detected. For bacterial vaginosis, 500 mg oral metronidazole (Flagyl) taken twice daily for 7 days and metronidazole intravaginal 0.75% gel were used. The recommended treatment for tichomoniasis was 2 g oral metronidazole (Flagyl) in a single dose. Oral azithromycin 1 g was scheduled for patients with *C. trachomatis* and a single oral 400-mg dose of cefixime was scheduled for patients with *N. gonorrhoeae.* The treatments were completed before oocyte pick-up.

When at least two leading follicles reached ≥18 mm in diameter, recombinant (rec) human Chorionic Gonadotropin (hCG Ovitrelle, Serono) was administered. Transvaginal ultrasound-guided oocyte retrieval was performed 34-36 hours after 0.25 mg rec hCG administration. Maximum 3 embryos were transferred on day 3 or 5 after oocyte retrieval. In both groups, good quality or blastocyst stage embryos were transferred. The luteal phase was supported with vaginal progesterone gel (Crinone 8%) administered daily starting on the day of oocyte pick-up.

Conception was defined as a positive hCG titer on day 12. We considered concentrations greater than 10 U/L as a positive result. Follow up ultrasound at 6 and 8 weeks of gestation confirmed clinical pregnancy. We followed all the women through to completion of the pregnancy.

The sample size of the study were calculated using G*Power (G*Power Ver. 3.00.10, Franz Faul, Üniversität Kiel, Germany) statistical package. The required sample size for 90% power, 5% Type I error, 10% Type II error and 20% effect size were calculated as 96 to better elucidate the impact of vaginal infection on pregnancy rates in intracytoplasmic sperm injection cycles. To protect the study from potential lost to follow-ups, we planned to enroll 120 patients.

The statistical analyses were performed using the Statistical Package for Social Sciences (version 11.5; SPSS Inc., Chicago). Descriptive data are expressed as mean ± standard deviation. Student’s t-test or Mann-Whitney U test were used for the comparison between groups. Pearson’s Chi-square and Fisher’s exact tests were used to analyze categorical data. The level of significance was set at p<0.05.

## RESULTS

One hundred twenty patients who met the inclusion criteria were enrolled into the study. Eighty-five patients (70.8%) who underwent IVF reached the embryo transfer stage. The mean age of the patients was 31±6.1 years. Clinical indications included male factor infertility in 56 (46.7%) cycles, ovulatory dysfunction in 24 (20.9%), unexplained infertility in 15 (12.5%), and tubal factor infertility in 6 (5%) cycles.

There was no significant difference between the ages of patients who conceived and those who did not conceive. Likewise, the two groups of patients were similar regarding the duration of infertility. Six of 25 women (24%) with unexplained infertility, 18 of 37 women (48.6%) with male factor infertility, and 6 of 23 (26.1%) women with other causes of infertility conceived successfully, but there was no difference among causes of infertility regarding the rates of pregnancy (p=0.077) (Table 1).

In group 1 (n=60), 45 patients underwent ET procedure. The rate of conception was 23.5% (n=4) in those with positive culture (n=17), and 42.9% (n=12) when no microorganism was detected (n=28). In group 2 (n=60), 40 patients reached ET. Of these 40 patients, conception was achieved in 35.0% (n=14). There was no significant difference between groups 1 and 2 in conception rates (p=0.96), and there was no difference among the groups of screen-positive, screen-negative, and not screened with culture, in terms of pregnancy success (p=0.42) (Table 2).

None of the patients had *N. gonorrhoeae* or *Trichomonas vaginalis.* Bacterial vaginosis was detected in 13 patients, and in 4 patients both bacterial vaginosis and *C. trachomatis* were recovered (Table 3).

Three of four patients who were screen-positive conceived and continued their pregnancy until a successful and healthy term delivery, but one spontaneously lost her pregnancy at six weeks of gestation. Eight of 12 patients who were screen-negative conceived and continued their pregnancy until a successful and healthy term delivery.

## DISCUSSION

We have shown that there was no difference betwen the groups of screen-positive, screen-negative and not screened in culture in terms of pregnancy success in our study population.

In previous studies, Chlamydial antibodies were found significantly higher in patients who attended clinics for tubal factor infertility and were candidates for IVF than patients who attended for other causes of female infertility^(10,11,12)^. Gaudoin et al.^(10)^ concluded that bacterial vaginosis and prior chlamydial infections had a significant association with tubal factor infertility, but were no associated with IVF outcomes in their study in 1999. However, in our study, these infections could not be accused for tubal factor infertility because the patients who were treated with IVF accounted for a small number. Chlamydial infections detected by favor of chlamydial antibodies were found not to effect IVF outcomes and embryonic implantation in a study by Osser et al.^(11)^ that included 121 women with tubal infertility. Also, in our study, chlamydial infections detected using EIA were found not to effect IVF outcomes.

Apart from this, several different studies investigated the effects of lower genital tract infections on female infertility. Spandorfer et al.^(13)^ studied the prevalence of bacterial vaginosis and abnormal bacterial vaginal microenvironment in infertile women, and cervical inflammatory cytokines caused by abnormal vaginal flora were found to be high in patients who presented Wilson with unexplained infertility; however, no effect was found on IVF outcomes^(14)^. In another study by Wilson et al.^(14)^ bacterial vaginosis was shown to be more frequent in patients undergoing IVF because of tubal infertility than in patients with other causes of infertility; these findings support the association among bacterial vaginosis, pelvic inflammatory disease, and tubal damage. Furthermore, in that study, bacterial vaginosis was detected more frequently in women undergoing IVF owing to anovulation, and hormones were shown to have effects on vaginal flora^(14)">^. Our study failed to show these effects because of the low number of patients. Ralph et al.^(15)^ found that bacterial vaginosis had no effect on pregnancy rates, but had abortifacient effects in the first trimester of pregnancy in their large study that included 867 infertile women who presented for IVF. Additionally, bacterial vaginosis was found to be associated with endometritis and preterm labor in a study by Korn et al.^(16)^.

The transmissive effects of bacterial vaginosis for sexually-transmitted diseases were studied by Yoshimura et al.^(17)^ with a sample of 406 patients. *C. trachomatis* was more frequently detected in patients with bacterial vaginosis than in those without. In the same study, young women were shown to be more inclined to bacterial vaginosis and sexually-transmitted diseases, especially Chlamydial cervicitis. In our study, there was no difference between the ages of women who were culture-positive and culture-negative. However, in our study, all patients who had Chlamydial infection also had bacterial vaginosis, and our results were similar with that study.

Wittemer et al.^(18)^ tried to determine the effects of treatment of vaginal and endocervical infections on IVF outcomes. The authors concluded that suspending the actual IVF cycle seemed more reasonable because of the possible deleterious effects of infection on embryonic implantation process, even if the patient was treated with a proper antimicrobial agent. Selim et al.^(19)^ stressed that women with bacterial vaginosis and with a decreased vaginal concentration of hydrogen peroxide-producing lactobacilli may have decreased conception rates and increased rates of failed pregnancy. Liversedge et al.^(20)^ stated that giving treatment for bacterial vaginosis before IVF could only be useful to lower the late pregnancy complications because there has been no confirmed effects of bacterial vaginosis on the rates of fertilization and implantation. However, in our study, there was no difference between the pregnancy rates of patients screened and not screened with culture, although lower pregnancy rates were detected in patients who were culture-positive.

The small number in our study group, and the subsequent scarcity of the patients who attended for IVF, and additionally, the unavailability of embryo transfer for every patient prevents us from being able to express the association between the types of infectious microorganisms and the etiology of infertility. A confirmatory culture was not taken after treatment. These represent the limitations of our study.

In conclusion, this study was performed to investigate whether endocervical and vaginal infections had any effects on pregnancy rates in IVF cycles. The patients who underwent endocervical and vaginal culture were compared with patients who had no culture. No significant difference was found between the patients who were sampled for culture and patients who were not sampled in terms of pregnancy rates. Also, no difference was found between patients who were culture-negative and those who were treated with antimicrobials after a culture-positive result.

## Figures and Tables

**Table 1:**
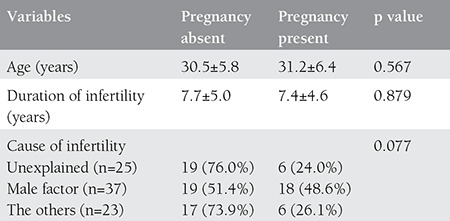
Distributions and comparisons of age, duration of infertility, and cause of infertility in patients who did and did not conceive

**Table 2:**
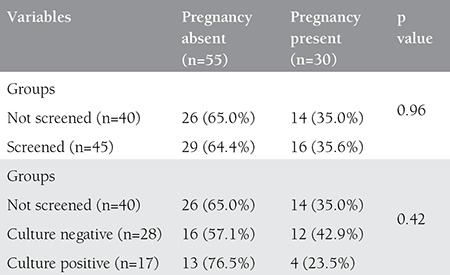
Screening with culture and its association with pregnancy rates

**Table 3:**
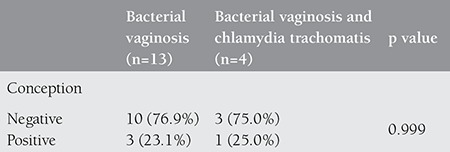
Conception rates according to the cultured microorganism
